# Hyperparathyroidism-induced secondary osteoporosis leading to recurrent non-traumatic vertebral compression fractures: A comprehensive case report

**DOI:** 10.1016/j.inpm.2023.100291

**Published:** 2023-11-10

**Authors:** Eric Paul Muneio, Akhil Chhatre, Nikhil Gopal, Clara Yuh, Kashif Hira, Pranamya Suri

**Affiliations:** a Department of Physical Medicine & Rehabilitation, Johns Hopkins Hospital, 1800 Orleans St, Baltimore, MD, 21287, USA; b Department of Transitional Medicine, Detroit Medical Center Sinai-Grace Hospital, 6071 Outer Dr W, Detroit, MI, 48235, USA; c Department of Physical Medicine & Rehabilitation, University of California, Irvine, 101 The City Dr S Building 3, Orange, CA, 92868, USA; d Department of Anesthesiology, HCA Florida Oak Hill Hospital, 11375 Cortez Blvd, Brooksville, FL, 34613, USA

## Abstract

**Background:**

Primary hyperparathyroidism, while increasing the susceptibility to osteoporosis, also amplifies the potential for fractures in vulnerable areas such as the femoral neck. It can also serve as an infrequent etiological factor behind vertebral compression fractures.

**Case report:**

This report discusses a case of multiple acute non-traumatic vertebral compression fractures in a patient diagnosed with primary hyperparathyroidism. The patient, a 79-year-old female with osteopenia (T Score −2.0, medically treated), had a history of left breast cancer treated with a partial mastectomy and radiation therapy. She presented with midline back pain resulting from T12 and L2 compression fractures and underwent balloon kyphoplasty. A week later, she reported severe low back pain, despite the absence of any new traumatic event. Repeat imaging showed multiple new, acute compression fractures at T10, T11, L1, and L3. Further workup revealed elevated parathyroid hormone levels and hypercalcemia, leading to a diagnosis of hyperparathyroidism.

**Conclusion:**

Multiple acute non-traumatic vertebral body compression fractures due to hyperparathyroidism is an uncommon clinical manifestation. This case emphasizes the need for an extended work-up of secondary osteoporosis in patients who experience multiple vertebral compression fractures.

## Abbreviation list

BMD: Bone Mineral Density
CASH: Cruciform Anterior Spinal Hyperextension Brace
CBC: Complete Blood Count
CMP: Comprehensive Metabolic Panel
ADLs: Activities of Daily Living
CT: Computed Tomography
DEXA: Dual-Energy X-ray Absorptiometry
MRI: Magnetic Resonance Imaging
ORIF: Open Reduction and Internal Fixation
PET: Positron Emission Tomography
PHPT: Primary Hyperparathyroidism
PTH: Parathyroid Hormone
TSH: Thyroid Stimulating Hormone

## 1 Introduction

Primary hyperparathyroidism increases the risk of osteoporosis and fractures [1]. Osteoporotic compression fractures constitute the majority of all vertebral compression fractures. In postmenopausal females, the estimated prevalence of these fractures is approximately 25 % [2]. These fractures are frequently treated conservatively with medications, bracing, and physical therapy. However, when pain is refractory to conservative care, vertebral augmentation may be required. Kyphoplasty is a widely accepted percutaneous procedure in which inflatable balloon tamps create a cavity within the fractured vertebral body. Cement is then inserted into the cavity to restore vertebral body height and reduce pain. Kyphoplasty has been proven to decrease pain and reduce disability [3].

The treatment and management of osteoporotic vertebral compression fractures encompass not only pain management but also treatment of the underlying cause. Osteoporosis remains the primary cause of vertebral compression fractures, with nearly 700,000 compression fractures occurring annually due to osteoporosis [2]. Hyperparathyroidism can induce secondary osteoporosis, which may be underdiagnosed in post-menopausal females. Parathyroid hormone is the chief regulator of calcium levels in extracellular fluid. Persistent exposure of bone to parathyroid hormone stimulates osteoclast action, leading to increased bone resorption and potentially exacerbating osteoporosis. Patients with primary hyperparathyroidism are found to have more than a three-fold increase in the risk of vertebral compression fractures [4].

Despite osteoporosis being the leading cause of compression fractures in the US, multiple non-traumatic compression fractures caused by hyperparathyroidism are much less common. This report introduces a patient who faced three distinct episodes of acute pain within a span of two months. Each episode heralded the onset of new compression fractures. Within a two-month period, the patient developed compression fractures at eight distinct vertebral levels. Notably, six of these fractures were effectively addressed with balloon kyphoplasty.

## 2 Case report

A 79-year-old female with a past medical history of osteopenia (T Score −2.0, medically Treated), left breast cancer (status post partial mastectomy and radiation therapy), type 2 diabetes mellitus, stage 3A chronic kidney disease, and fibromyalgia, presented with intractable low back pain in the absence of recent trauma. While recovering from an ankle fracture that was managed with open reduction and internal fixation (ORIF), she experienced an acute exacerbation of her back pain.

At an outside institution, her persistent and severe low back pain, unrelated to recent trauma, suggested possible nerve root irritation or inflammation. As part of the treatment strategy frequently used in the management of chronic pain conditions related to spinal etiologies, she was administered epidural steroid injections aiming to target the source of inflammation and provide immediate anti-inflammatory effects and pain relief. Unfortunately, the epidural steroid injections did not ameliorate her symptoms. Following this, she was given two Medrol (methylprednisolone) dose packs to manage potential flare-ups. However, her pain persisted despite these interventions, along with trials of cyclobenzaprine and hydrocodone-acetaminophen.

**Fig. 1:**
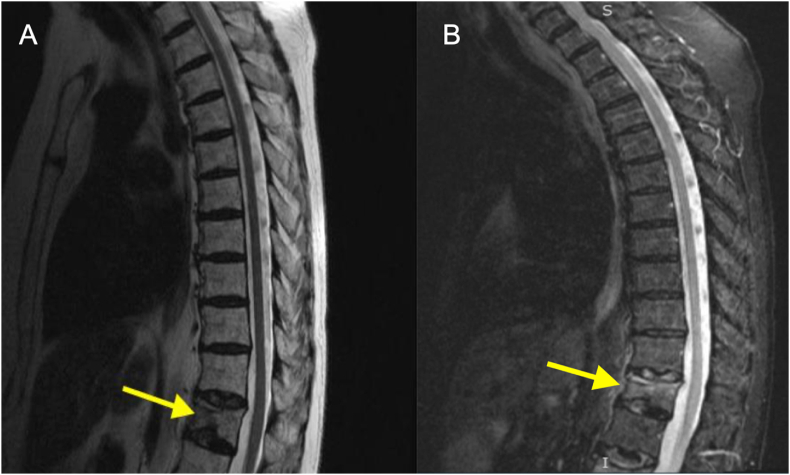
T12 vertebral compression fracture visualized using MRI sequences. (A) T2-weighted MRI with an arrow pointing to the fracture site. (B) STIR sequence with an arrow highlighting the bright signals indicative of edema/inflammation at the corresponding fracture location.

**Fig. 2:**
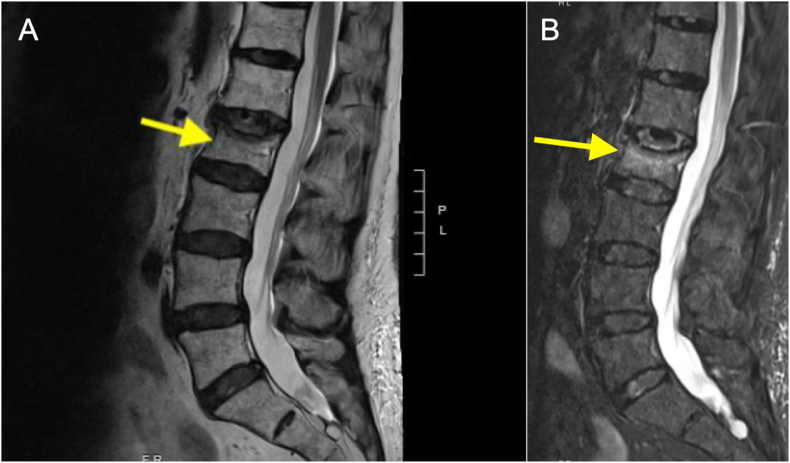
L2 vertebral compression fracture visualized using MRI sequences. (A) T2-weighted MRI with an arrow pointing to the fracture site. (B) STIR sequence with an arrow highlighting the bright signals indicative of edema/inflammation at the corresponding fracture location.

Subsequently, the patient sought care at the emergency department for her unrelenting severe low back pain. The pain was midline, radiated to the right buttock, and was not associated with focal weakness or sensory changes. On physical examination, tenderness was elicited in the lower thoracic and upper lumbar spine regions. Initial CT scans of the abdomen and pelvis performed in the emergency department showed no evidence of compression fractures. However, a subsequent MRI of the thoracic and lumbar spine revealed acute T12 and L2 compression fractures, which had undergone a 40 % reduction in height and exhibited signs of bone marrow edema (Fig. 1, Fig. 2). Notably, no bony mass or retropulsion was detected.

Given the MRI findings and the refractory nature of her symptoms, the patient was referred to our interventional spine practice. Here, a kyphoplasty was considered due to the severity of her radiological findings and her lack of response to more conservative treatment modalities. It is noteworthy to mention that the patient had previously been on Fosamax and Prolia for osteoporosis. These treatments were effective in ameliorating her condition to osteopenia, as evidenced by her latest DEXA scan results (T Score −2.0). After careful deliberation, a decision was reached to undertake a balloon kyphoplasty at the T12 and L2 levels, utilizing a unipedicular approach.

**Fig. 3:**
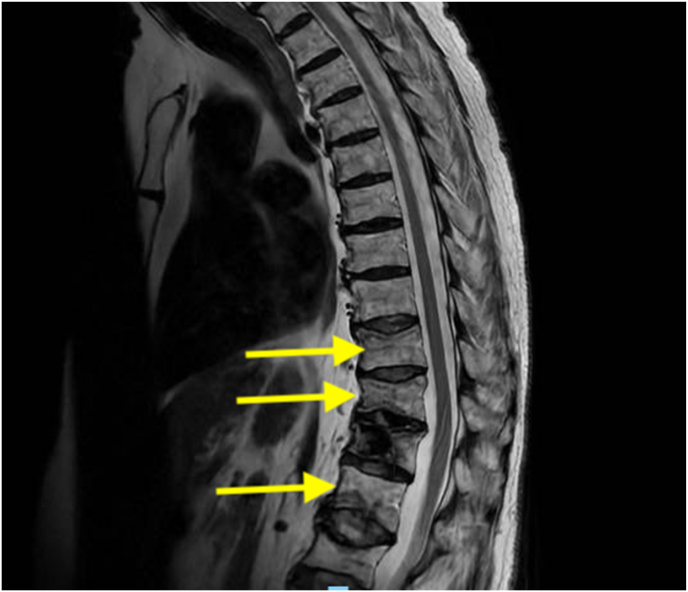
T2-weighted MRI sequence illustrating compression fractures at T10, T11, and L1 levels, each highlighted by arrows. T12 demonstrates post-kyphoplasty changes.

The patient initially experienced mild to moderate post-procedure pain, which was managed with muscle relaxants and oxycodone 5mg–10mg. However, a week after the procedure, she reported severe lower back pain, which had begun spontaneously. A CASH brace was ordered, and the patient was compliant with its use. However, she continued to experience relentless and unceasing pain. Repeat MRI scans of the thoracic and lumbar spine were ordered, revealing new posterior body compression fracture deformities of T10 and T11 with mild anterior/central wedging, and acute compression deformities of L1 and L3 without significant retropulsion (Fig. 3).

Given the presence of multiple acute compression fractures, tests for vitamin D, calcium, PTH, TSH, CBC, and CMP for further evaluation were ordered for further evaluation. A malignancy workup was also initiated given her history of breast cancer. The PET scan did not reveal any malignancy. Laboratory results revealed elevated parathyroid hormone levels and hypercalcemia, leading to a diagnosis of hyperparathyroidism. The patient was referred to endocrinology for further treatment of her hyperparathyroidism. The multiple compression fractures occurring in the context of osteopenia suggested the presence of a bone disease due to primary hyperparathyroidism. The patient is scheduled to undergo a parathyroidectomy to treat the primary hyperparathyroidism.

**Fig. 4:**
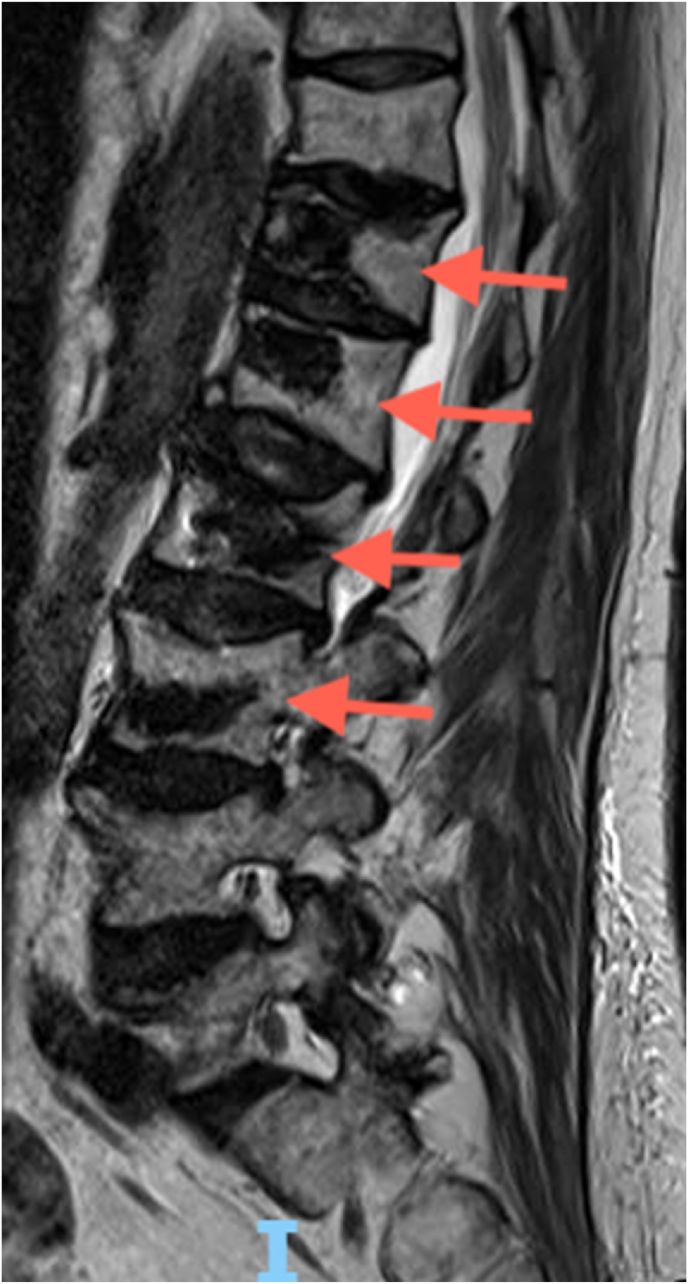
T2-weighted MRI sequence illustrating post-kyphoplasty changes at T12, L1, L2, and L3 levels, indicated by orange arrows. (For interpretation of the references to colour in this figure legend, the reader is referred to the Web version of this article.)

**Fig. 5:**
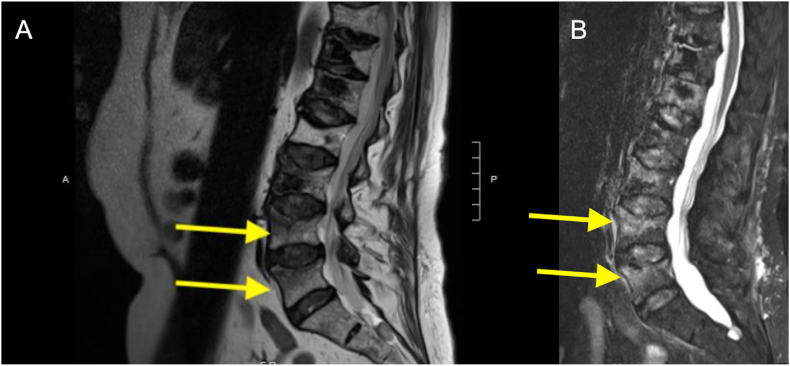
New L4 and L5 12 vertebral compression fractures visualized using MRI sequences. (A) T2-weighted MRI with an arrow pointing to the fracture site. (B) STIR sequence with an arrow highlighting the bright signals indicative of edema/inflammation at the corresponding fracture location.

At this time, most of her pain was localized to the lumbar spine, and the decision was

made to treat the L1 and L3 fractures with balloon kyphoplasty using a unipedicular approach (Fig. 4). After the kyphoplasty of the L1 and L3 vertebral compression fractures, her pain significantly improved. However, shortly after the second kyphoplasty, she again experienced acutely worsened low back pain, and a repeat MRI was ordered to assess for any additional fractures. The MRI demonstrated new, acute L4 and L5 compression fractures (Fig. 5). She was once again treated with L4 and L5 balloon kyphoplasty using a unipedicular approach. After the procedure, she reported significant relief from her acute low back pain. In total, the patient developed compression fractures at eight distinct vertebral levels due to hyperparathyroidism. Six of these fractures were treated with kyphoplasty.

## 3 Discussion

Our report highlights an unusual manifestation of primary hyperparathyroidism (PHPT), where despite being under treatment for osteoporosis and showcasing improved T scores, the patient developed multiple vertebral compression fractures within a remarkable short time span. PHPT, characterized by an unregulated overproduction of parathyroid hormone (PTH), often causes a decrease in bone mineral density (BMD), leading to osteoporosis and heightened fracture risk [5].

The parathyroid glands maintain calcium homeostasis in the body, a vital process for various physiological functions. PTH enhances bone resorption by stimulating osteoclast action. Chronic exposure to PTH, as seen in PHPT, disrupts the equilibrium between bone formation and resorption, tilting the balance toward resorption and eventually causing bone loss and osteoporosis [6]. Thus, the underlying PHPT in our patient might have contributed to the rapid progression of osteoporosis and resultant compression fractures.

The impact of PHPT on the skeletal system is often underestimated. PHPT-induced osteoporosis, despite being a significant health concern, is frequently underdiagnosed [7]. This could be attributed to the prevalence of normocalcemic PHPT, a phenotype that presents with normal serum calcium levels despite increased PTH, complicating the diagnosis [8]. While our patient was diagnosed with primary hyperparathyroidism, it's vital to consider secondary hyperparathyroidism in patients with a history of chronic oral or inhaled steroid use. Glucocorticoid exposure can lead to inhibited calcium absorption from the gastrointestinal tract and impact renal tubular calcium reabsorption, potentially causing secondary hyperparathyroidism [9]. However, in our patient's case, the recent, brief steroid exposure is considered unlikely to result in hyperparathyroidism.

The rapid transition of our patient's diagnosis from osteopenia to multiple vertebral compression fractures, all within a concise temporal frame, underscores the ramifications of unchecked PHPT-induced osteoporosis. This progression merits a heightened index of suspicion for PHPT in patients presenting with an avalanche of vertebral compression fractures, especially if they are refractory to osteoporosis treatment.

In our patient's case, the magnitude of her pain and the significantly positive response to each preceding kyphoplasty necessitated further intervention. Each kyphoplasty provided the patient with significant initial pain relief prior to re-fracturing. Despite the occurrence of new fractures, kyphoplasty was continually opted for due to the patient's functionally limiting pain which significantly impaired her ability to ambulate and carry out ADLs. Moreover, while conservative care options like bracing, pain medications, and physical therapy are commonly beneficial for managing post-compression fracture symptoms, in this particular case, kyphoplasty emerged as the preferred choice based on the severity of her symptoms, her functional impairment, and her positive response to previous interventions.

A therapeutic cornerstone in our case was balloon kyphoplasty. This intervention, with proven efficacy in managing vertebral compression fractures among osteoporotic patients, was deployed for every fracture our patient incurred [10]. Kyphoplasty involves the inflation of a balloon tamp within the fractured vertebra to create a cavity which is then filled with bone cement. This procedure has been shown to alleviate pain, restore vertebral body height, and improve patient mobility [11]. In our case, kyphoplasty served as a pivotal intervention in relieving the pain associated with the patient's recurrent vertebral fractures.

Furthermore, it's pertinent to discuss the potential association between vertebroplasty and subsequent vertebral fractures. Kyphoplasty, while effective, is known to increase vertebral stiffness [12]. This alteration in vertebral dynamics has been suggested to elevate the risk of adjacent level fractures. Moreover, factors such as kyphosis angle and disc degeneration, evident in our patient as Pfirrmann grade II-III, can further contribute to adjacent level fractures following vertebral augmentation [12]. The combination of brittle bones due to the patient's underlying condition, along with these changing mechanical forces, might have culminated in a ‘perfect storm’ scenario leading to multiple fractures in our patient.

The literature on this topic offers varied perspectives. Some early research indicates a potential increased risk, yet recent studies, including a 2018 meta-analysis, lean toward no augmented risk [13,14]. It's also noteworthy that the specific context of hyperparathyroidism has yet to be extensively explored in relation to subsequent vertebral fractures. A more thorough investigation of compression fractures in patients with hyperparathyroidism could elucidate the incidence of subsequent fractures in this cohort.

Addressing the fundamental cause is a critical aspect of osteoporotic vertebral fractures management. This case emphasizes the importance of identifying and managing concurrent PHPT in patients presenting with osteoporosis accompanied by compression fractures. Despite an improvement in bone mineral density with the ongoing osteoporosis medical treatment, the patient in this case sustained multiple fractures. Notably, these fractures were non-traumatic in nature, underscoring the severe fragility of the patient's bones and prompting further investigation. Upon discovering elevated PTH and hypercalcemia, the management strategy had to be revised to target the PHPT.

A multidisciplinary approach, encompassing not only interventional treatments such as kyphoplasty but also surgical and medical therapy, is warranted for managing osteoporosis due to PHPT. For patients resistant to medical therapy or presenting with complications such as fractures, parathyroidectomy may be necessary. Moreover, the use of antiresorptive agents such as alendronate has been found beneficial in these patients, significantly increasing BMD and reducing fracture risk [5].

In conclusion, our case report illustrates the diverse presentations of PHPT, from normocalcemic to osteoporotic manifestations with vertebral compression fractures. It underscores the importance of considering PHPT as a potential differential in patients with osteoporosis refractory to treatment. A comprehensive approach, including kyphoplasty for symptomatic fracture management and appropriate medical or surgical intervention for PHPT, is critical for optimal patient outcomes.

## Declaration of competing interest

The authors declare that they have no known competing financial interests or personal relationships that could have appeared to influence the work reported in this paper.
